# A rare complication of vascular surgery – subclinical esophageal perforation secondary to supra-aortic debranching and TEVAR: a case report

**DOI:** 10.1097/RC9.0000000000000777

**Published:** 2026-08-11

**Authors:** András Herczeg, Veronika Papp, Ákos Balázs, Attila Szijártó, Zoltán Szeberin, Tamás Vass

**Affiliations:** a Department of Surgery, Transplantation and Gastroenterology, Semmelweis University, Budapest, Hungary; b Department of Vascular and Endovascular Surgery, Heart and Vascular Centre, Semmelweis University, Budapest, Hungary

**Keywords:** case report, esophagus, perforation, reconstruction, TEVAR

## Abstract

**Introduction and importance:**

Combined supra-aortic debranching and thoracic endovascular aortic repair (TEVAR) are well-established therapeutic options for complex aortic arch pathologies. Although rare, late complications such as esophageal injury can be life-threatening and require prompt multidisciplinary management. Several cases of delayed esophageal perforation complicated by mediastinitis have been reported in the literature. However, perforation presenting solely as a graft infection causing microembolizations remains exceedingly rare.

**Case presentation:**

A 56-year-old male patient with a history of multiple prior cardiovascular surgeries (including TEVAR and zone 1 debranching) and no other relevant medical or habitual risk factors was admitted with recurrent fever, painful ischemic petechiae of the left hand, and left-sided transient ischemic attacks. Imaging studies demonstrated infection of the carotico-subclavian polytetrafluoroethylene (PTFE) crossover graft. Esophagogastroduodenoscopy revealed esophageal perforation caused by erosion of the retroesophageal PTFE graft. Complete graft explantation, arterial homograft reconstruction, and surgical repair of the esophageal lesion were performed. Subsequently, the patient required partial graft resection due to pseudoaneurysm formation of the homograft, as well as percutaneous angioplasty for stenosis of the subclavian anastomosis. At present, the patient remains in a satisfactory and stable condition.

**Clinical discussion:**

Retroesophageally positioned grafts may lead to esophageal perforation through chronic mechanical compression and subsequent ischemic necrosis of the esophageal wall. In the present case, the graft itself appeared to tamponade the perforation, thereby limiting mediastinal contamination and contributing to the subclinical presentation.

**Conclusion:**

The present case highlights the potential for an atypical and insidious clinical presentation that may delay diagnosis. Early graft explantation and appropriate reconstruction may lead to favorable clinical outcomes.

## Introduction

Since its first clinical use in 1992, thoracic endovascular aortic repair (TEVAR) has become a widely adopted first-line therapy for both acute and chronic aortic diseases. The true incidence of associated complications is likely underestimated due to the paucity of long-term follow-up data; however, rates of secondary interventions and reoperations have been reported to range between 16–32% and 0.4–7.9%, respectively^[^1^]^. Although most complications are vascular in nature (e.g., endoleak or graft migration), gastrointestinal adverse events, such as aorto-esophageal fistula and esophageal perforation, have also been described^[^2^]^. Esophageal perforation is an uncommon but life-threatening complication that is associated with high mortality and may arise from multiple etiological mechanisms, including direct compression of the esophagus or occlusion of its segmental arterial supply by the aortic stent graft. Subclinical presentation of TEVAR-associated esophageal perforation that allows for elective management is exceedingly rare.

HIGHLIGHTS

- Gastrointestinal complications following thoracic endovascular aortic repair are rare but potentially life-threatening.
- Adhesion between the graft and the esophageal wall may limit mediastinal contamination.
- A multidisciplinary approach may enable organ-preserving surgical management with favorable outcomes.

This case report has been reported in line with the SCARE checklist^[^3^]^.

## Case presentation

**Figure 1.**
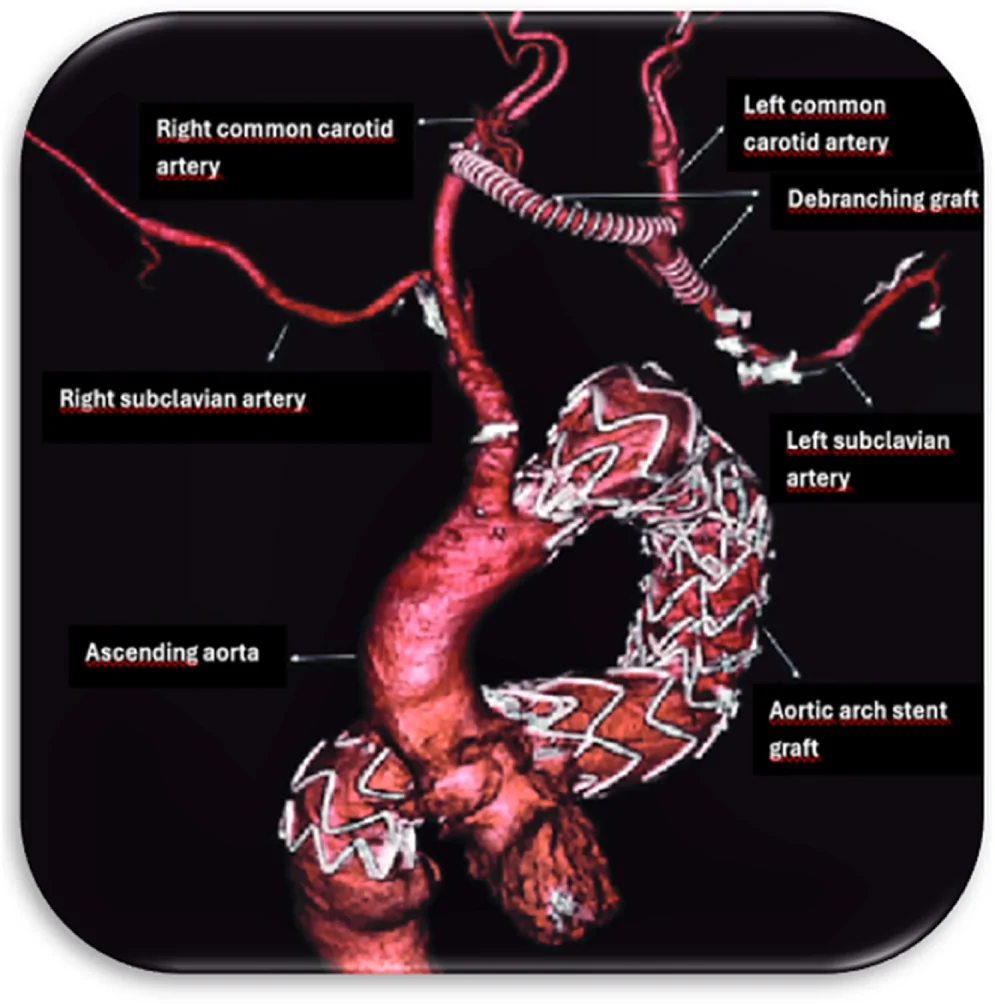
Zone 1 debranching and TEVAR with a right common carotid-to-left subclavian bypass and left common carotid reimplantation.

A 56-year-old male patient with no other relevant medical or habitual risk factors had a history of multiple prior cardiovascular surgeries (descending aortic patch repair for coarctation, TEVAR and Zone 1 debranching for a symptomatic pseudoaneurysm at the site of the patch repair, coronary bypass and mitral valve replacement). During the TEVAR and debranching surgery, a retroesophageal right common carotid–left subclavian polytetrafluoroethylene (PTFE) bypass graft was created, along with reimplantation of the left common carotid artery, followed by the deployment of the aortic stent graft to Zones 3-4 (Fig. 1).

**Figure 2.**
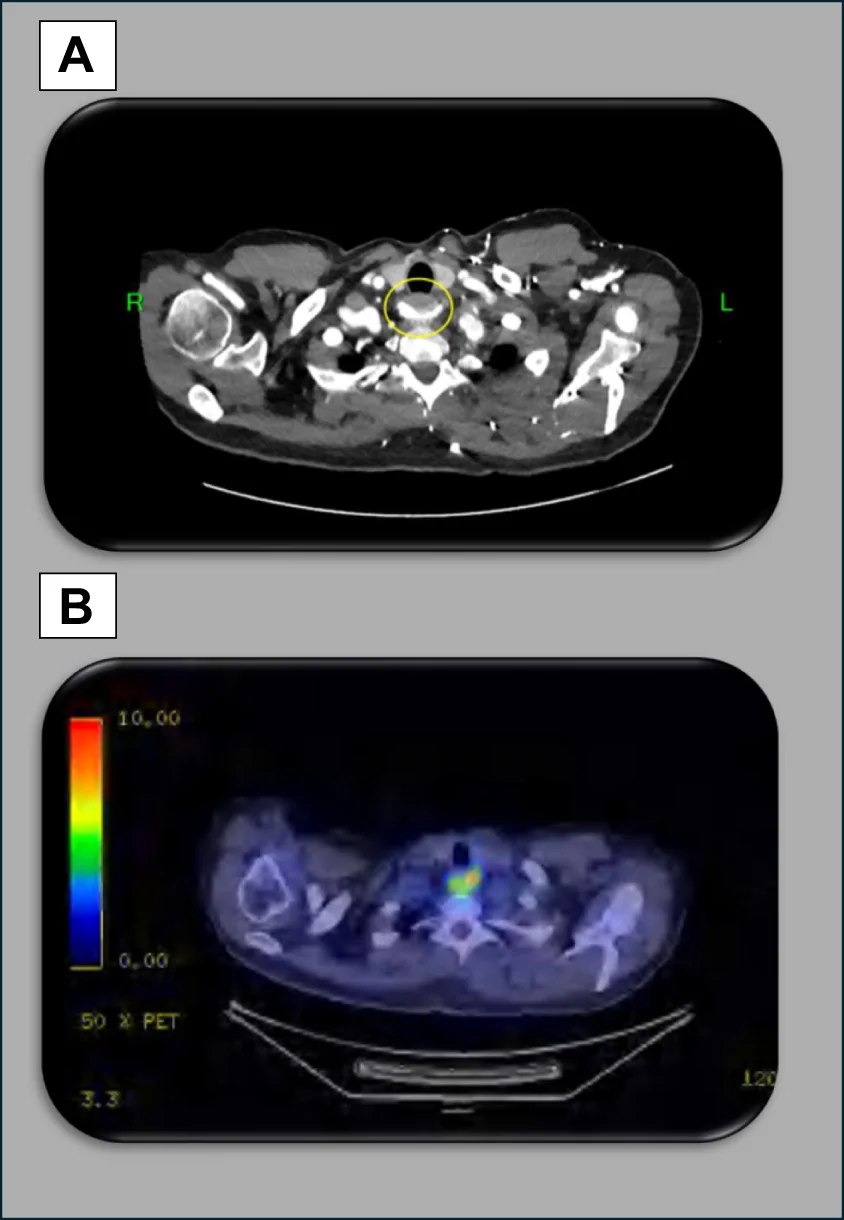
**(A)** Schematic 3D illustration of the retroesophageal PTFE graft (yellow circle). **(B)** Increased tracer uptake on PET-CT, consistent with graft infection.

**Figure 3.**
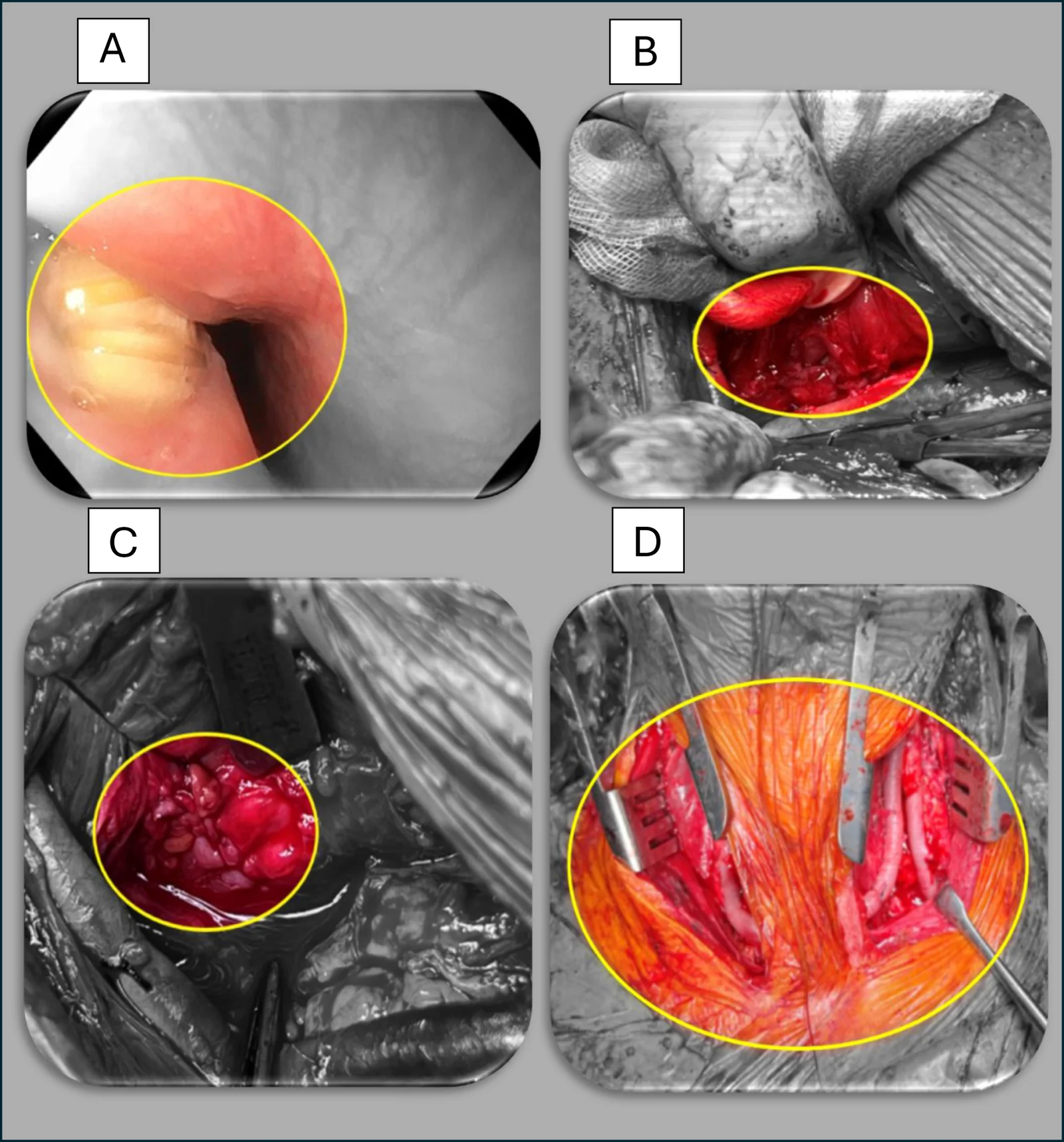
**(A)** Endoscopic view of the PTFE graft visible through the esophageal perforation. **(B)** Intraoperative view of the perforated esophagus. **(C)** Two-layer reconstruction of the esophageal wall. **(D)** Pretracheal carotico-carotid crossover and carotico-subclavian bypass using a femoral artery homograft.

One year later, the patient presented as an outpatient with recurrent episodes of high fever, left upper limb pain, ischemic petechiae, and transient ischemic attack (presenting as temporary right-hand weakness). Laboratory testing demonstrated elevated inflammatory markers, including leukocytosis and increased C-reactive protein levels, indicating systemic infection. In accordance with current European guidelines for the evaluation of suspected vascular graft infection, PET-CT was performed and demonstrated increased metabolic activity at the graft site extending toward the esophagus, consistent with graft infection and raising suspicion of esophageal involvement (Fig. 2). Subsequent esophagogastroduodenoscopy confirmed a perforation located 16 cm from the incisors and measuring approximately 10 mm in diameter, which appeared to be tamponaded by the graft, preventing mediastinal leakage (Fig. 3A). During the same endoscopic session, a percutaneous endoscopic gastrostomy (PEG) was placed to ensure adequate nutritional support as part of preoperative management. Blood cultures were positive for *Eikenella corrodens, Haemophilus parainfluenzae*, and *Streptococcus anginosus*; therefore, targeted antimicrobial therapy with ceftriaxone and gentamicin was initiated. Apart from the aforementioned symptoms, the patient remained free of severe septic manifestations, allowing for consideration of elective reconstruction following multidisciplinary discussion, in accordance with current European guidelines on the management of vascular infections^[^4^]^. The reconstructive procedure included complete removal of the infected retroesophageal PTFE graft, creation of a pretracheal carotico-carotid crossover and carotico-subclavian bypass using a femoral artery homograft (Fig. 3D). Biological reconstruction using a homograft was preferred in the setting of infection to reduce the risk of reinfection. The esophageal perforation was visualized (Fig. 3B) and repaired with a two-layer continuous suture (Fig. 3C)

Postoperatively, the patient developed transient right-sided hemiparesis without radiologic evidence of acute ischemia. Persistent left recurrent laryngeal nerve paresis required corticosteroid therapy and subsequent phoniatric rehabilitation, resulting in satisfactory recovery of vocal function without dyspnea. A follow-up contrast swallow study demonstrated no evidence of esophageal leakage, allowing gradual reintroduction of oral feeding and subsequent removal of the PEG. The patient was discharged in good general condition on postoperative day 32. At outpatient endoscopic follow-up, the esophagus was found to be completely healed, with no residual lesion.

During follow-up, several additional vascular interventions became necessary. Four weeks after the homograft bypass, a bovine pericardial tube graft was implanted at the distal end of the homograft due to a 2 × 2 cm pseudoaneurysmal degeneration. One year later, significant stenosis of the subclavian anastomosis necessitated percutaneous stent implantation. At the 27-month follow-up, the patient remains in stable condition with satisfactory swallowing and vocal function and no signs of recurrent infection; ongoing surveillance includes annual CTA imaging.

## Discussion

Although rare, gastrointestinal complications following TEVAR represent serious and potentially life-threatening events associated with significant morbidity and mortality. This often results from delayed diagnosis and consequent mediastinitis requiring major esophagectomy. Similar insidious presentations across surgical fields may pose significant diagnostic challenges and delay appropriate management^[^5^]^. Yaguchi *et al* reported seven cases of esophageal perforation after TEVAR, with a mortality rate of 85.7%^[^6^]^. In this case, esophageal erosion and perforation were caused by the retroesophageal bypass graft rather than the TEVAR itself, without direct aortic–esophageal communication, likely due to chronic compression and gradual erosion of the esophageal wall. The presence of gastrointestinal and oral flora pathogens in prior blood cultures, together with *Candida albicans* isolated from the explanted graft, supports the hypothesis of graft infection secondary to esophageal flora translocation. Dysphagia has been reported under similar circumstances; however, a tamponaded esophageal perforation represents an extreme and exceptionally rare presentation^[^7^]^. Adhesion between the esophageal wall and the PTFE graft limited local inflammation, resulting in a subclinical presentation and enabling organ-preserving management (i.e., primary repair instead of esophagectomy). This case demonstrates that graft infection and septic microembolization may be the presenting manifestations of a contained esophageal perforation. These findings underscore the importance of maintaining a high index of suspicion for atypical presentations in patients with prior complex aortic interventions.

## Conclusion

Retroesophageal placement of vascular grafts may lead to esophageal perforation due to chronic compression and subsequent erosion of the esophageal wall. This case highlights the potential for an atypical and insidious clinical presentation that may delay diagnosis. In selected cases, early graft explantation and appropriate reconstruction within a multidisciplinary team, including vascular surgery, general surgery, and gastroenterology, can result in favorable outcomes.

## Data Availability

The data are not publicly available due to patient privacy restrictions but are available from the corresponding author upon reasonable request.
